# Lung cancer metabolism: a dynamic and adaptive network

**DOI:** 10.1007/s00018-026-06417-5

**Published:** 2026-10-19

**Authors:** Sara Gholamhosseinian-Najjar, Christian Frezza

**Affiliations:** 1 Institute for Metabolomics in Ageing, Cluster of Excellence Cellular Stress Responses in Aging-associated Diseases (CECAD), Faculty of Medicine and University Hospital Cologne, University of Cologne, Cologne, Germany; 2 Institute of Genetics, Cluster of Excellence Cellular Stress Responses in Aging-associated Diseases (CECAD), Faculty of Mathematics and Natural Sciences, University of Cologne, Cologne, Germany; 3 Center for Molecular Medicine (CMMC), University of Cologne, Cologne, Germany

**Keywords:** Lung cancer metabolism, Metabolic plasticity, Glucose metabolism, Glutamine metabolism, Nucleotide metabolism, Sulphur metabolism.

## Abstract

Lung cancer remains the leading cause of cancer-related mortality worldwide. Although substantial progress has been made in defining the genetic, epigenetic, and molecular drivers of this heterogeneous disease, metabolic reprogramming has only recently emerged as a major determinant of tumour progression and therapeutic response. Cumulative evidence demonstrates that rather than relying on a single nutrient source or pathway, cancer cells integrate glucose and glutamine, fatty acid and one-carbon metabolism, as well as nucleotide biosynthesis, and redox-supporting circuits in a highly context-dependent manner. These programs vary across histological and molecular subtypes, reflecting the influence of lineage identity and oncogenic state on metabolic organisation. In both non-small cell lung cancer and small cell lung cancer, subtype-specific constraints shape distinct metabolic states, yet a common feature is the ability to rapidly shift between alternative nutrients and biosynthetic routes. This adaptability supports continued growth under nutrient limitation, maintains redox homeostasis, and contributes to resistance against conventional and targeted therapies. By summarising current mechanistic evidence, this review presents lung cancer metabolism as an integrated and adaptable system rather than a collection of isolated pathways, and identifies metabolic dependencies that may be leveraged for therapeutic intervention.

## Introduction

Lung cancer remains the leading cause of cancer-related death, accounting for 1.8 million deaths among 2.5 million diagnosed cases [1]. Unfortunately, it is also the cancer type with the largest number of potentially preventable cases worldwide, with a marked sex-specific pattern. Among women, an estimated 477,869 cases could have been prevented, mainly by reducing smoking (60.5%), followed by air pollution (27.5%) and occupational exposures (12.0%). In men, the burden is substantially higher, with 1,326,453 preventable cases, again driven primarily by smoking (69.4%), then air pollution (15.8%), and occupational exposures (14.7%) [2]. Beyond the issue of late diagnosis, lung cancer management is further complicated by marked biological and clinical heterogeneity, which influences treatment selection and contributes to variable outcomes.

Non-small cell lung cancer (NSCLC) accounts for approximately 85–90% of all lung cancer cases and is mainly composed of lung adenocarcinoma (LUAD) and lung squamous cell carcinoma (LUSC), each of which exhibits a distinct molecular architecture and therapeutic susceptibility [3–5]. In LUAD, some of the most frequent driver alterations include epidermal growth factor receptor (*EGFR*), serine-threonine kinase 11 (*STK11*), Kirsten rat sarcoma viral oncogene homolog (*KRAS*), Kelch-like ECH-associated protein 1 (*KEAP1*), and tumour protein p53 (*TP53*), and together these alterations account for most of the molecular diversity observed in this subtype [3]. LUSC likewise displays a characteristic mutational profile, with recurrent alterations in *TP53*, fibroblast growth factor receptor (*FGFR*), phosphatase and tensin homolog (*PTEN*), and phosphatidylinositol-4,5-bisphosphate 3-kinase catalytic subunit alpha (*PIK3CA*), reflecting both subtype-specific biology and partial overlap with LUAD, particularly through shared disruptions in *TP53* [4, 5].

Small cell lung cancer (SCLC), which accounts for the remaining 10–15% of cases, is biologically and clinically distinct from NSCLC and is defined in nearly all tumours by concurrent loss of function of *TP53* and retinoblastoma 1 (*RB1*) [6]. Beyond this obligate genomic backbone, SCLC is further organized into molecular and lineage-based subtypes that reflect distinct transcriptional programs. Classical neuroendocrine (NE) tumours are characterized by high expression of achaete-scute homolog 1 (*ASCL1*), whereas non-neuroendocrine (non-NE) tumours show dominant MYC activity and reduced *ASCL1* expression. Importantly, SCLC also displays remarkable plasticity during progression and under therapy pressure, with *MYC* upregulation contributing to subtype switching and the emergence of alternative states marked by neurogenic differentiation factor 1 (*NEUROD1*) or POU class 2 homeobox 3 (*POU2F3*) expression, thereby reinforcing treatment escape and disease evolution [6–9]. Although these genomic alterations help explain many of the differences in prognosis and treatment response across different lung cancer subtypes, they do not fully capture the adaptive capacity of the disease, especially under therapeutic pressure and microenvironmental stress.

Despite substantial therapeutic progress in lung cancer, durable disease control remains elusive for many patients. Especially in SCLC, treatment is still anchored in platinum-based chemotherapy, most commonly paired with etoposide. Radiotherapy is used in limited-stage disease and immunotherapy is incorporated in selected extensive-stage settings, while surgery is reserved for only a very small subset of early lesions. Although these regimens can produce rapid initial responses, relapse is frequent, reflecting the aggressive clinical course of SCLC, its pronounced genomic instability, and its ability to adopt alternative cellular states associated with treatment resistance [10]. In contrast, NSCLC has moved toward a more personalized treatment paradigm, in which surgery, radiotherapy, chemotherapy, targeted therapy, and immunotherapy are selected according to stage, histology, and molecular features. However, even in this biomarker-driven setting, resistance and disease recurrence remain as major barriers to long-term benefits [11, 12]. These limitations indicate that genetic alterations, while essential to understanding lung cancer biology, are not sufficient on their own to explain therapeutic failure, thereby highlighting the need for a deeper understanding of the disease from multiple aspects, including metabolism.

Although it has long been recognised that tumour cells reprogram their metabolism in response to environmental stress and to fuel growth, survival, and metastasis, this area of cancer biology has been overlooked for many years. Recently, however, increasing attention has been directed toward the metabolic state of cancer cells, particularly NSCLC, to better understand how malignant cells preserve growth and even thrive under conditions such as hypoxia, nutrient deprivation, mechanical stress, and oxidative damage. A growing body of evidence now shows that lung cancer cells rely on metabolic flexibility as a fundamental survival strategy. By remodelling interconnected metabolic networks, they can sustain energy production, support biomass accumulation, maintain redox balance, and withstand therapy-induced stress. These adaptive programs are increasingly viewed as active contributors to tumour progression, metastasis, and treatment resistance, making metabolic plasticity a defining hallmark of lung cancer biology [13–15].

In this review, we examine the major metabolic programs that lung cancer cells exploit to sustain growth, survive stress, and evade treatment. We first examine glucose metabolism, which supports adenosine triphosphate (ATP) generation and provides intermediates for biosynthesis, with tumour cells often increasing glucose uptake and glycolytic flux to meet their energy demands and adapt to hypoxia. We then consider fatty acid synthesis and β-oxidation, which together determine how cancer cells acquire, store, and oxidise lipids to further sustain energy production and maintain membrane integrity. We next discuss glutamine metabolism, a key source of carbon and nitrogen that fuels the tricarboxylic acid (TCA) cycle, redox homeostasis, and nucleotide synthesis, making it especially important for proliferating and therapy-resistant cells. The section on serine and glycine metabolism highlights how cancer cells use this pathway to generate nucleotides and antioxidant capacity, thereby supporting DNA synthesis and cellular adaptation to oxidative stress. The nucleotide metabolism section addresses how lung cancer cells increase nucleotide production and salvage pathways to sustain rapid proliferation and repair damage under therapeutic pressure. Finally, we cover sulphur metabolism, including the methionine cycle, transsulfuration (TSS), cysteine use, and glutathione (GSH) production, which collectively help tumour cells maintain redox balance and resist oxidative injury. In addition, we briefly discuss emerging metabolic approaches in clinical intervention and diagnosis, although these applications remain limited and require further development before they can be broadly integrated into routine patient care. Together, these pathways illustrate how lung cancer rewires central metabolism to support malignant progression and create dependencies that may be therapeutically exploitable. Of note, due to space limitations, we decided to focus on cancer cell-intrinsic metabolic features, leaving aside the interesting aspects of metabolic changes in the tumour microenvironment and immune cells more generally.

## Glucose metabolism as a central node in lung cancer progression

Glucose is the primary carbon source for most cells, providing both energy and metabolic intermediates required for biosynthesis and redox balance. In tumour cells, glucose demand is often elevated to sustain rapid proliferation, support anabolic growth, and maintain survival under stress, making glucose metabolism a central feature of cancer metabolic reprogramming. Tumours also exploit glucose to generate precursors for nucleotide, amino acid, and lipid synthesis, as well as to produce nicotinamide adenine dinucleotide phosphate (NADPH) and other metabolites that help buffer oxidative stress.

**Fig. 1:**
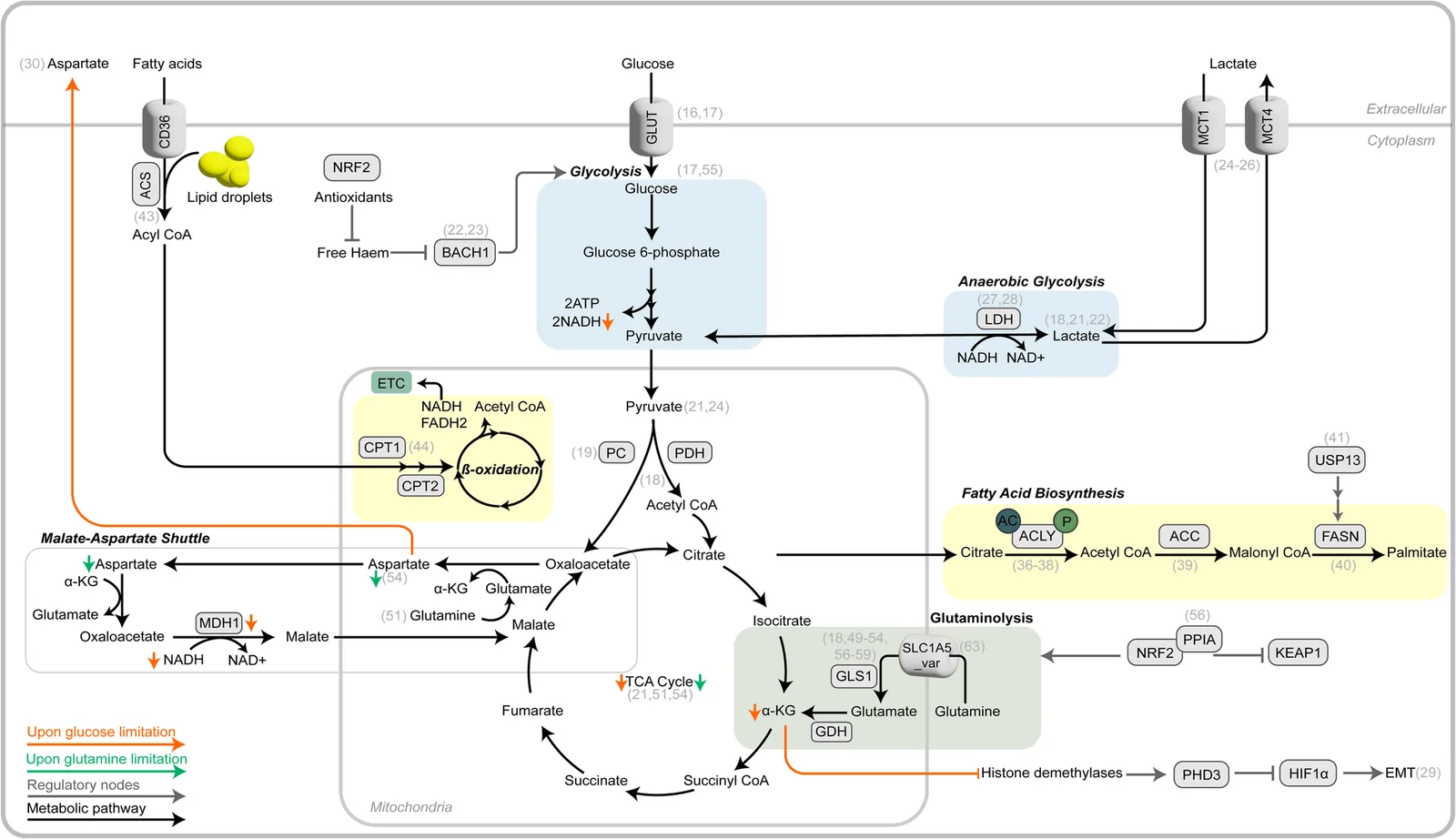
Metabolic landscape of glucose, glutamine, and fatty acid metabolism in lung cancer**.** Glycolysis converts glucose to pyruvate, which is either routed into the TCA cycle to support OXPHOS or reduced to lactate that can be exported and reused as a fuel (blue). Glutamine, the second major carbon donor, undergoes glutaminolysis to generate α-KG and replenish TCA intermediates (green); depletion of glucose or glutamine can disrupt the MAS and truncate mitochondrial anaplerosis. In parallel, surplus citrate is exported to the cytoplasm and used for de novo fatty acid synthesis, while β-oxidation of fatty acids provides an alternative source of acetyl-CoA and reducing equivalents to meet energetic needs (yellow). Key regulators such as NRF2, USP13, and PPIA modulate pathway fluxes in response to nutrient availability and cellular stress. ACC: acetyl-CoA carboxylase; ACLY: ATP-citrate lyase; ACS: acyl-CoA synthetase; α-KG: alpha-ketoglutarate; ATP: adenosine triphosphate; BACH1: BTB and CNC homology 1; CD36: cluster of differentiation 36; CPT1: carnitine palmitoyltransferase 1; CPT2: carnitine palmitoyltransferase 2; ETC: electron transport chain; FASN: fatty acid synthase; FDH: formate dehydrogenase; GLS1: glutaminase 1; GLUT: glucose transporter; GDH: glutamate dehydrogenase; HIF1α: hypoxia-inducible factor 1 alpha; LDH: lactate dehydrogenase; MAS: malate-aspartate shuttle; MDH1: malate dehydrogenase 1; MCT1: monocarboxylate transporter 1; MCT4: monocarboxylate transporter 4; NRF2: nuclear factor erythroid 2-related factor 2; OXPHOS: oxidative phosphorylation; PC: pyruvate carboxylase; PDH: pyruvate dehydrogenase; PPIA: peptidylprolyl isomerase A; SLC1A5: solute carrier family 1 member 5; USP13: ubiquitin-specific peptidase 13; TCA: tricarboxylic acid cycle; acetyl-CoA: acetyl coenzyme A; NADH: nicotinamide adenine dinucleotide, reduced form; NAD+: nicotinamide adenine dinucleotide, oxidized form; FADH2: flavin adenine dinucleotide, reduced form

Glycolysis begins with the uptake of glucose via glucose transporters (GLUTs), an entry point that tumour cells commonly utilise but differentially modulate (Fig. 1). In particular, *GLUT1* expression in LUSC is associated with enhanced glucose uptake and glycolytic flux that renders these cells specifically susceptible to GLUT1 inhibition [16], while in LUAD, its upregulation correlates with poorer overall survival [17]. Upon uptake, glucose enters the two-phase glycolytic pathway. In the first phase, the energy-investment phase, two ATP molecules are consumed to phosphorylate and rearrange the six-carbon glucose molecule, which is subsequently cleaved into two three-carbon molecules. In the second phase, the energy-payoff phase, these three-carbon intermediates are oxidised, generating two pyruvate molecules and a net yield of two ATP and two reduced nicotinamide adenine dinucleotides (NADH) per glucose molecule. In LUAD, increased expression of key glycolytic enzymes such as glyceraldehyde-3-phosphate dehydrogenase (GAPDH) and phosphoglycerate kinase 1 (PGK1) are linked to reduced overall survival, highlighting the clinical relevance of glycolytic reprogramming [17]. The produced pyruvate serves as a critical metabolic junction that directs glucose-derived carbon into multiple downstream pathways. Under aerobic conditions, pyruvate enters mitochondria and undergoes two distinct fates: it can be oxidatively decarboxylated by pyruvate dehydrogenase (PDH) to generate acetyl-CoA for TCA cycle entry, or it can be carboxylated by pyruvate carboxylase (PC) to produce oxaloacetate through ATP-dependent anaplerosis. In lung cancer, isotope-tracing studies have shown substantial heterogeneity in the relative contribution of PDH- and PC-mediated carbon entry into the TCA cycle [18]. Importantly, PC-mediated anaplerosis appears to be selectively enhanced in early-stage NSCLC and is essential for tumour survival and proliferation [19]. Under anaerobic conditions, or in certain cancer cells favouring aerobic glycolysis, pyruvate is instead reduced to lactate by lactate dehydrogenase (LDH), regenerating NAD + to maintain glycolytic flux [20]. Consistent with this, metabolomic and gene-expression analyses across NSCLC models have linked hypoxia-associated transcriptional programs to elevated lactate secretion, although NE-like cells release less lactate per glucose molecule consumed, again underscoring subtype-specific metabolic behaviour [18].

A landmark longitudinal 11-year clinical study using intraoperative ¹³C-glucose infusions in NSCLC patients revealed that aggressive tumours exhibit elevated glucose oxidation into TCA cycle intermediates, distinguishing malignant from benign lesions and predicting poor survival outcomes. Tumours showed markedly higher ¹³C enrichment in pyruvate, lactate, and TCA metabolites compared with adjacent healthy lung tissue. Elevated TCA cycle labelling correlated with worse overall and recurrence-free survival regardless of age, sex, histology, or genetic mutations. Metastatic tumours demonstrated even greater TCA cycle labelling than primary NSCLC. Patient-derived xenografts (PDXs) maintained the same metabolic labelling patterns, whereas metastatic PDXs achieved 67% engraftment compared with only 7.5% for primary tumours, demonstrating that metabolic characteristics could predict metastatic potential. Single-cell RNA sequencing analysis confirmed that tumour epithelial cells express the highest levels of oxidative phosphorylation (OXPHOS) genes among all tumour cell types, identifying them as the main drivers of oxidative metabolism. In the PDX model, pharmacological inhibition of complex I in the electron transport chain (ETC) reduced TCA cycle labelling and decreased metastatic burden in the lung, brain, and lymph nodes while leaving primary tumour growth unchanged, thus demonstrating that OXPHOS is specifically essential for metastatic progression rather than primary tumour proliferation [21].

These findings on glucose-driven TCA cycle metabolism and OXPHOS-dependent metastasis appear to complement rather than contradict earlier studies on BTB domain and CNC homolog 1 (BACH1)-mediated glycolytic activation. Two independent studies revealed that BACH1 stabilisation, triggered by either dietary antioxidants (N-acetylcysteine and vitamin E) or endogenous nuclear factor erythroid 2-related factor 2 (NRF2) activation, promotes lung cancer metastasis through glycolytic reprogramming. Both pathways reduce free haem levels, stabilising BACH1, which transcriptionally upregulates hexokinase 2 (*HK2*) and *GAPDH*, increasing glucose uptake, glycolysis rates, and lactate secretion to fuel metastatic progression [22, 23]. These seemingly discordant metabolic programs likely reflect context-dependent mechanisms and temporal relationships in lung cancer progression. Early-stage tumours may rely on BACH1-driven glycolysis to support redox homeostasis and nucleotide synthesis. Metastatic tumours, however, may shift toward glucose oxidation into the TCA cycle and OXPHOS to meet the energy demands of invasive spread. Metabolic plasticity allows tumours to utilise glycolysis for biomass production while simultaneously engaging TCA cycle oxidation for energy production during metastasis. Thus, BACH1-mediated glycolysis may support primary tumour growth and survival under reduced oxidative stress, while glucose-TCA-OXPHOS coupling drives the energetics of metastatic dissemination, potentially representing sequential metabolic adaptations rather than mutually exclusive pathways.

In line with these findings, glucose oxidation appears to be the main source of carbon in less perfused regions of the tumour, whereas alternative carbon sources, particularly lactate, are preferentially utilised in better-perfused areas, highlighting the importance of the microenvironment in shaping cellular metabolism [24]. This metabolic flexibility depends on the expression of monocarboxylate transporter 1 (MCT1), the primary lactate importer in tumour cells, enabling cancer cells to import lactate as an energy substrate [25]. In highly glycolytic cells, blocking MCT4, the lactate exporter, causes intracellular lactate accumulation and triggers reactive oxygen species (ROS)-dependent apoptosis [26] (Fig. 1). Consistent with the importance of lactate as a fuel and metabolic node, lactate dehydrogenase B (LDHB) was identified as a critical therapeutic target in KRAS-driven NSCLC. Epithelial-like tumour-initiating NSCLC cells depend on LDHB to convert lactate back to pyruvate to fuel OXPHOS in mitochondria. In these cells, *LDHB* silencing induces severe mitochondrial dysfunction characterised by impaired respiratory chain activity, OXPHOS failure, and persistent DNA damage affecting both mitochondrial and nuclear DNA, as evidenced by elevated histone H2AX (γH2AX) phosphorylation. Mechanistically, LDHB deficiency depletes essential metabolites, including TCA cycle intermediates, amino acids, and nucleotides from both purine and pyrimidine biosynthesis pathways. Among these, nucleotide depletion appears to serve as the primary driver of anti-tumourigenic effects, as nucleotide supplementation partially rescues the survival and sphere-formation defects of *LDHB*-silenced cells [27, 28].

Aberrant glucose utilisation is a hallmark of cancer, yet therapeutic approaches targeting glucose starvation have shown limited success, mainly because tumour metabolic plasticity triggers a multifaceted reprogramming response that enables adaptation to nutrient deprivation. A key mechanism underlying this resistance is metabolic-epigenetic coupling triggered by glucose deprivation. Reduced glucose availability lowers α-ketoglutarate (αKG) levels, which in turn diminishes the activity of αKG-dependent histone demethylases and promotes histone hypermethylation. This epigenetic shift can drive cellular dedifferentiation through dysregulation of the histone methyltransferase EZH2, leading to suppression of prolyl-hydroxylase 3 (*PHD3*) and subsequent stabilisation of hypoxia-inducible factor 1α (HIF1α). The resulting pseudohypoxic state promotes epithelial-to-mesenchymal transition (EMT), a process associated with poor prognosis in human LUAD [29] (Fig. 1). In parallel, glucose deprivation triggers conserved metabolic adaptation in lung cancer cells, bronchial epithelial cells, and cancer-associated fibroblasts, leading to accumulation and export of aspartate. Mechanistically, glucose scarcity depletes NADH, limiting the NADH-dependent function of cytoplasmic malate dehydrogenase 1 (MDH1) and truncating the malate-aspartate shuttle (MAS). As a consequence, aspartate synthesized from glutamine via glutamic-oxaloacetic transaminase 2 (GOT2) is redirected for export rather than purine synthesis [30]. Within the tumour microenvironment, glucose deprivation can induce tumour cells to release leukemia inhibitory factor (LIF), an interleukin-6 (IL-6) family cytokine, leading to defective N-glycosylation and enhanced angiogenesis [31]. These coordinated responses represent a comprehensive adaptive strategy that lung cancer cells employ to survive nutrient stress and maintain proliferative capacity.

Overall, lung cancer cells treat glucose as far more than a simple energy source. Rather, they use it flexibly to support growth, maintain redox balance, and adapt to hypoxia and nutrient stress. Importantly, glucose metabolism in lung cancer is highly context-dependent, as different subtypes, stages, and microenvironmental regions can favor distinct glucose fates, ranging from glycolytic flux and lactate handling to mitochondrial oxidation and anaplerotic support. This metabolic plasticity also extends beyond metabolism itself, influencing transcriptional and epigenetic programs that promote dedifferentiation, invasion, and resistance. Taken together, lung cancer cells continually reconfigure glucose metabolism to match environmental demands, making it a central and adaptable framework for tumour progression, metastasis, and therapeutic escape.

## The lipid metabolic circuit: balancing de novo synthesis and β-oxidation

Fatty acids are central lipids that serve both as structural components of membranes and as dense energy stores. Their metabolism is built around two complementary pathways: (1) de novo fatty acid biosynthesis, which generates long‑chain fatty acids to support membrane biogenesis, lipid droplet formation, and the production of lipid‑derived signalling molecules, and (2) β‑oxidation, which breaks down stored and imported fatty acids to yield intermediates for ATP production and redox balance. These opposing processes are tightly coordinated and often inversely regulated, allowing cells to shift between lipid storage and lipid‑derived energy, depending on nutrient availability, metabolic demand, and cellular state. Keeping this balance is essential for maintaining energy homeostasis and adapting to feeding, fasting, and stress conditions [32–34].

In cancer, this balance is rewired toward a dynamic lipid network that supports tumour growth and stress adaptation; de novo synthesis is upregulated to fuel biosynthetic demands, while β‑oxidation is engaged to provide a flexible, efficient energy source from both endogenous and exogenous fatty acids [32–34]. This reprogramming is often orchestrated by glucose availability and citrate‑driven supply of cytosolic acetyl‑CoA, creating a metabolic continuum from glycolysis to lipogenesis. As glucose fuels glycolytic flux and mitochondrial acetyl-CoA accumulates, part of this acetyl-CoA is exported as citrate to support de novo fatty acid synthesis in the cytosol. In this process, acetyl-CoA generated from pyruvate condenses with oxaloacetate to form citrate, which is transported to the cytosol and cleaved by ATP-citrate lyase (ACLY) to regenerate acetyl-CoA. Cytosolic acetyl-CoA is then carboxylated by acetyl-CoA carboxylase (ACC) to malonyl-CoA, the committed and rate-limiting step requiring biotin and ATP. Both acetyl-CoA and malonyl-CoA are next transferred onto acyl-carrier protein (ACP) to form acetyl-ACP and malonyl-ACP, which feed into the fatty acid synthesis cycle, where fatty acid synthase (FASN) catalyses the NADPH-dependent assembly of palmitate as the principal long-chain saturated end product of de novo lipogenesis [35] (Fig. 1).

The flow from glycolysis to fatty acid synthesis is tightly controlled at the level of ACLY, which acts as a central metabolic hub integrating multiple post-translational inputs. ACLY activity is tightly regulated by acetylation and phosphorylation, which together govern its stability and lipogenic output. Under high-glucose conditions, acetylation of ACLY at lysines 540, 546 and 554 (3 K) increases ACLY stability by blocking ubiquitylation at these sites, thereby enhancing de novo lipid synthesis, cell proliferation, and tumour growth; elevated 3 K acetylation is observed in human lung cancers [36]. In addition to the 3 K cluster, ACLY harbours regulatory acetylation sites at K17 and K86, where Rho guanine nucleotide exchange factor 3 (ARHGEF3) engages ACLY and promotes its association with the cytosolic deacetylase sirtuin 2 (SIRT2). Modulation of ACLY acetylation at these residues via the ARHGEF3-SIRT2 axis preserves ACLY protein abundance, increases acetyl-CoA supply and fatty acid synthesis, and sustains NSCLC proliferation and tumour growth [37]. Acetylation control at both the 3 K and K17/K86 sites therefore positions ACLY as a central metabolic hub in lung cancer, with multiple post-translational circuits converging on continued lipogenesis and tumour progression. Separately, phosphorylation of ACLY has also been shown to activate the enzyme and drive malignant progression in NSCLC; both total ACLY and its phosphorylated form (p-ACLY) are upregulated in lung tumours compared with normal lung, and high p-ACLY correlates with advanced disease and poor survival, highlighting phosphorylation as a clinically relevant regulatory layer [38].

Following ACLY, the pathway is further constrained by ACC1, the rate‑limiting enzyme of fatty acid synthesis, which is selectively upregulated in NSCLC, as these cells appear to be dependent on its enzymatic activity for proliferation and tumour growth. Genetic *Acc1* deletion or pharmacologic inhibition with the allosteric inhibitor, ND‑646, nearly abolishes glucose‑derived fatty acid synthesis, triggers ER stress and apoptosis, and markedly impairs NSCLC cell viability, all of which can be rescued by exogenous palmitate supplementation [39]. Thus, once acetyl‑CoA is made and exported, the pathway is governed by ACC1 and FASN, converting it into a functional lipid pool that fuels membrane biogenesis and signalling. This dependency is especially pronounced in KRAS‑driven LUAD, which is functionally linked to a lipogenic program centred on FASN and a distinct extracellular signal-regulated kinase 2 (ERK2)‑biased MAPK signature. In this model, oncogenic KRAS induces *FASN* expression and de novo fatty acid synthesis, producing a specific lipidomic and proteomic profile that distinguishes KRAS‑mutant tumours from non‑KRAS subtypes. Specifically, KRAS preferentially activates ERK2, whereas ERK1 is more prominent in non‑KRAS lung cancers. Due to this dependency, pharmacologic inhibition of FASN blocks proliferation of KRAS‑associated tumour cells [40]. In SCLC, the same lipogenic node is reinforced by post‑translational stabilisation. FASN is stabilised by the deubiquitinase USP13, which is highly expressed and correlated with poor prognosis. Loss of USP13 impairs cancer stem cell properties, tumour growth, and chemoresistance, effects that are blunted when *FASN* is downregulated or inhibited pharmacologically with TVB‑2640 [41]. Thus, across LUAD and SCLC, FASN emerges as a shared effector of oncogenic lipid metabolism.

Complementing de novo lipogenesis, both intracellular and extracellular lipid sources function as a major energy reservoir that can be mobilised through mitochondrial β‑oxidation when glucose or other fuels are limited. In this process, fatty acids are either taken up into cells via transport proteins such as cluster of differentiation 36 (CD36), or derived from intracellular sources such as lipid droplets (Fig. 1). Fatty acids funnelled for oxidation are turned into acyl‑CoA by acyl‑CoA synthetase (ACS), committing them to oxidative catabolism. Next, long‑chain acyl‑CoAs are imported into the mitochondrial matrix by the carnitine shuttle, in which carnitine palmitoyltransferase 1 (CPT1) on the outer membrane forms acylcarnitine, and carnitine palmitoyltransferase 2 (CPT2) regenerates acyl‑CoA in the matrix. There, the acyl‑CoA enters the β‑oxidation pathway, which progressively shortens the fatty acid while producing acetyl‑CoA, NADH, and FADH₂ that feed the TCA cycle and ETC to support ATP generation [42]. In this way, the lipogenic machinery that builds fatty acids is balanced by a parallel oxidative apparatus that can burn them when needed, allowing tumours to switch between storage and energy production according to nutrient availability. This balance is actively rewired in KRAS-driven NSCLC, where Acyl‑CoA synthetase long‑chain family member 3 (*ACSL3*), a member of the ACS family, is upregulated, which leads to enhanced fatty acid uptake and β‑oxidation for ATP production. In KRAS‑driven mouse and xenograft models, *Acsl3* suppression decreases tumour growth and increases apoptosis [43]. In parallel, ASCL1^high^ SCLC cells rely on mitochondrial fatty acid catabolism to fuel OXPHOS. Pharmacologic inhibition of CPT1, the rate‑limiting transporter for mitochondrial β‑oxidation, selectively impairs proliferation of ASCL1‑dominant cells, demonstrating that β‑oxidation is a functional vulnerability in this subtype [44].

Together, these findings illustrate how lipid metabolism in lung cancer is not a static background process but a dynamic, tightly balanced network; de novo fatty acid synthesis builds and stores a lipid pool that supports membrane biogenesis, signalling, and stress tolerance, while mitochondrial β‑oxidation repeatedly mobilises both intracellular and extracellular fatty acids to generate energy and maintain redox balance when glucose or other fuels are limited. This functional interplay between lipid accumulation and lipid catabolism allows tumours to flexibly adapt to changing nutrient conditions and underpins their growth and survival. This reciprocal dependency argues for therapeutic strategies that disrupt both arms, for instance, combining FASN inhibition with CPT1 blockade, rather than either alone.

## Glutamine: a key carbon and nitrogen donor for lung cancer progression

Cancer cells consume large amounts of glucose in the tumour microenvironment to produce ATP rapidly but inefficiently, which can reduce glucose availability up to 10-fold compared with healthy tissue microenvironments and create an energy crisis [45, 46]. To compensate, tumour cells rewire their metabolism to exploit alternative nutrients, among which glutamine stands out as one of the most efficient and cost-effective energy and biosynthetic substrates. Beyond its role as an ATP source, glutamine donates nitrogen for amino acid and nucleotide synthesis, supports redox balance via GSH production, and provides carbon intermediates that feed key metabolic pathways.

In mitochondria, glutamine is catabolised to glutamate by glutaminase 1 (GLS1), which is then converted to α-KG by glutamate dehydrogenase (GDH), enabling carbon entry into the TCA cycle [47]. This glutamine-derived α-KG can either flow through the TCA cycle and ultimately produce ATP, a process called glutaminolysis, or it can be reductively carboxylated to produce citrate and acetyl-CoA for fatty acid synthesis [48] (Fig. 1).

NSCLC cells often rely on glutaminolysis to sustain the TCA cycle, ATP production, and mitochondrial function [18, 49]. The importance of GLS1 is revealed under glucose-limited conditions, where RAS signalling induces mitochondrial cytochrome *c* oxidase 2 (*MT-CO2*), a core component of ETC complex IV, thereby promoting *GLS1* expression and enhancing glutaminolysis. This metabolic shift allows tumour cells to reduce their reliance on glucose and instead use glutamine as a major carbon source for survival and growth [50]. When *GLS1* is inhibited or knocked out, all TCA cycle metabolites are reduced, and the ATP pool is depleted [51], leading to cell cycle arrest [51, 52]. Oncogenic MYC further reinforces this dependency by upregulating *GLS1* through inhibiting its repressors, miR-23a and miR-23b [53]. In addition to fuelling the TCA cycle, GLS1 supports ATP production by supplying glutamate for the MAS, thereby enabling transfer of cytosolic reducing equivalents into mitochondria; accordingly, GLS1 inhibition impairs MAS function and contributes to ATP depletion [51]. Under glutamine starvation, reduced TCA cycle flux also limits mitochondrial aspartate export via the aspartate-glutamate carrier SLC25A12 (AGC1), lowering cytosolic aspartate availability and promoting cell death. In line with this, combined inhibition of SLC25A12 and GLS1 suppresses tumour growth in vivo by simultaneously blocking aspartate export through the MAS and glutamine-derived carbon entry into the TCA cycle [54]. However, the relative contribution of glutamine may be context-dependent. Indeed, in vivo metabolic tracing studies have demonstrated that glucose, rather than glutamine, may provide the major carbon input to the TCA cycle in some lung tumours, highlighting the strong influence of the tissue microenvironment on cancer metabolism [55].

In KRAS-driven NSCLC, mutations frequently occur in *KEAP1*, the negative regulator of NRF2. When KEAP1 is lost, NRF2 becomes hyperactivated, boosting antioxidant defences, driving tumour cell proliferation and inducing expression of a wide spectrum of target genes, including krueppel-like factor 5 (*KLF5*) and *SLC1A5* [56] (Fig. 1). Beyond protecting cells from oxidative stress, overactive NRF2 remodels cellular metabolism by shifting glucose and glutamine utilisation toward the synthesis of new biomolecules. Specifically, NRF2 increases glucose uptake and directs glucose-derived carbon into the pentose phosphate pathway (PPP) to generate nucleotides, while also elevating glutamine uptake and channelling it into the TCA cycle to replenish intermediates needed for lipid and protein synthesis. This metabolic shift creates a cellular environment that supports rapid growth and anabolic activity. Research has shown that genes involved in glutaminolysis are critical for the survival of these NRF2-hyperactive cells. When glutamine is removed or the glutamine transporter, SLC1A5, is blocked, these cancer cells lose their ability to proliferate effectively. The metabolic changes driven by NRF2 ultimately disrupt normal carbon metabolism, making NRF2-hyperactivated tumours particularly vulnerable to glutaminase inhibitors, a promising therapeutic strategy for this aggressive cancer subtype [57–59]. The discovery that peptidylprolyl isomerase A (PPIA) acts as a key stabilizer of NRF2 has opened up new therapeutic possibilities for such cancer types with hyperactivated NRF2. PPIA binds directly to NRF2, preventing KEAP1 from tagging NRF2 for ubiquitination, therefore allowing NRF2 accumulation. The FDA-approved drug cyclosporin A (CsA) hinders the PPIA-NRF2 interaction [56] by forming a complex with PPIA1 itself [60]. Consequently, this interaction interferes with glutamine metabolism mediated by the NRF2-KLF5-SLC1A5 pathway. When combined with a GLS inhibitor, it shows synergistic efficacy by significantly inhibiting tumour progression in NRF2-hyperactivated NSCLC patient-derived xenograft models [56].

**Fig. 2:**
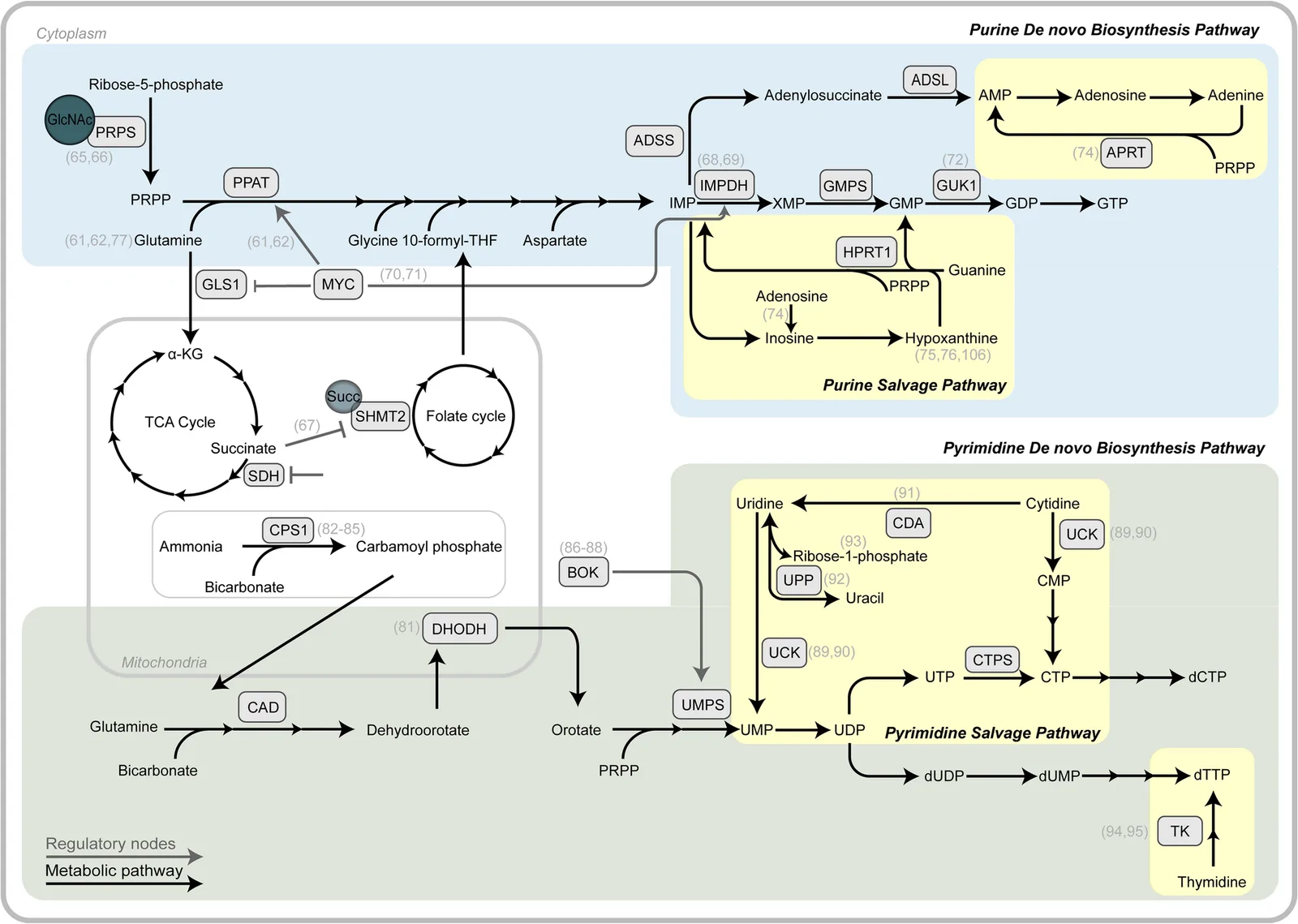
Nucleotide metabolism in lung cancer. De novo purine biosynthesis begins with PRPS-mediated formation of PRPP, the ribose donor required for ring assembly, and proceeds to IMP production, which serves as the branch point for AMP and GMP synthesis (blue). Nucleotide pools are further maintained through salvage of catabolised nucleotides and nucleobases by HPRT1 and APRT (yellow). Pyrimidine biosynthesis initiated by the CAD protein relies on mitochondrially localized DHODH to generate orotate, which is utilised for UDP synthesis. UDP then serves as an intermediate for CTP and dTTP synthesis (green). In parallel, the ammonia detoxification pathway is exploited to supply carbamoyl phosphate for pyrimidine synthesis. Pyrimidine pools are maintained through salvage of cytidine, uridine and thymidine, by enzymatic reaction of UCK and TK, respectively. MYC and BOK modulate purine and pyrimidine metabolism, respectively. APRT: adenine phosphoribosyltransferase; ADSL: adenylosuccinate lyase; ADSS: adenylosuccinate synthetase; BOK: BCL2-related ovarian killer; CAD: carbamoyl-phosphate synthetase 2, aspartate transcarbamylase, and dihydroorotase; CDA: cytidine deaminase; CPS1: carbamoyl phosphate synthetase 1; CTPS: CTP synthase; dCTP: deoxycytidine triphosphate; DHODH: dihydroorotate dehydrogenase; dUDP: deoxyuridine diphosphate; dUMP: deoxyuridine monophosphate; dTTP: deoxythymidine triphosphate; GLS1: glutaminase 1; GMPS: GMP synthetase; GUK1: guanylate kinase 1; GlcNAc: N-acetylglucosamine; HPRT1: hypoxanthine-guanine phosphoribosyltransferase 1; IMPDH: inosine monophosphate dehydrogenase; MYC: MYC proto-oncogene; PPAT: phosphoribosylamine–glycine ligase; PRPS: phosphoribosyl pyrophosphate synthetase; PRPP: phosphoribosyl pyrophosphate; SDH: succinate dehydrogenase; SHMT2: serine hydroxymethyltransferase 2; Succ: succinate; TK: thymidine kinase; UCK: uridine-cytidine kinase; UMP: uridine monophosphate; UMPS: uridine monophosphate synthetase; UDP: uridine diphosphate; UTP: uridine triphosphate; α-KG: alpha-ketoglutarate; UPP: uridine phosphorylase

Beyond serving as a carbon source, glutamine also provides nitrogen for biosynthetic pathways, including nucleotide synthesis. This nitrogen-donating function becomes particularly important in the malignant progression of SCLC tumours, where MYC-driven upregulation of phosphoribosyl pyrophosphate amidotransferase (*PPAT*) enhances the flux of glutamine-derived nitrogen toward nucleotide biosynthesis rather than glutaminolysis through GLS1 [61]. Similarly, upregulation of *PPAT* in NSCLC was associated with poor prognosis and aggressive progression [62] (Fig. 2).

Taken together, these studies show that glutamine metabolism is highly context-dependent, with its contribution shaped by tumour type, oncogenic drivers, nutrient availability, and the surrounding microenvironment. As a versatile carbon and nitrogen donor, glutamine supports TCA cycle anaplerosis, biosynthetic reactions, nucleotide production, and redox homeostasis, making it a central nutrient for tumour growth and survival. At the same time, this dependence is not uniform across all lung tumours, as some cells rely more heavily on glutamine than others, depending on their metabolic state and environmental constraints. Even so, inhibiting glutamine transport at both the plasma membrane (SLC1A5) and mitochondrial (SLC1A5_var) levels has shown therapeutic promise by limiting nutrient supply and impairing the replicative capacity of tumour cells [63, 64]. The therapeutic implication is that glutaminase inhibition will likely work best in stratified populations where tumours are profiled for glutamine dependency.

## Nucleotide metabolism in lung cancer

The high proliferative rate of cancer cells generates a sustained demand for nucleotides to support DNA replication, RNA synthesis, and ribosome biogenesis. To meet this requirement, cancer cells engage multiple nucleotide-producing routes. Collectively, the available evidence indicates that nucleotide metabolism in lung cancer is not governed exclusively by either de novo synthesis or salvage pathways. Instead, tumour cells exhibit a high degree of metabolic plasticity, dynamically deploying both de novo and salvage pathways in ways dictated by nutrient availability, oncogenic genotype, and cellular metabolic state.

### De novo and salvage metabolism of purines

At the entry point of de novo purine synthesis, ribose-5-phosphate, derived from the PPP, is converted to its active form and the ribose donor for purine ring assembly, phosphoribosyl pyrophosphate (PRPP), by phosphoribosyl pyrophosphate synthetase 1 (PRPS1) (Fig. 2). In lung cancer, PRPS1 is selectively activated by O-GlcNAc transferase (OGT)-dependent O-GlcNAcylation, which leads to its hexamerization. This sustained PRPS1 activity enhances flux through the de novo purine synthesis pathway [65, 66]. Enhanced PRPS1 O-GlcNAcylation in human lung cancer tissue samples has been associated with tumour growth and chemotherapy resistance [66]. Commitment to purine nucleotide synthesis begins with the catalytic activity of PPAT, the next enzyme in the de novo purine pathway. PPAT incorporates the amide nitrogen of glutamine into phosphoribosyl pyrophosphate to form phosphoribosylamine. Selective upregulation of *PPAT* redirects glutamine flux toward purine biosynthesis, rather than toward the TCA cycle via glutaminolysis, and has been associated with disease progression and malignant transformation in NSCLC and SCLC, respectively [61, 62]. Once a cell commits to de novo purine synthesis, the purine ring is built stepwise on the ribose scaffold of PRPP through nine enzymatic reactions. This pathway draws on several key inputs, including glycine, aspartate, the amide nitrogen of glutamine, one-carbon units carried by 10-formyl-tetrahydrofolate (10-formyl-THF), and CO₂, ultimately producing inosine monophosphate (IMP), the first fully formed purine nucleotide (Fig. 2). Because mitochondrial one-carbon metabolism supplies formate for this process, disruption of the mitochondrial folate cycle can limit IMP production by reducing the availability of one-carbon donors. In line with this, exogenous formate can rescue IMP synthesis in lung cancer cells with impaired folate metabolism. The importance of this pathway is further highlighted by evidence that inhibition of succinate dehydrogenase (SDH) causes succinate accumulation, which promotes succinylation of serine hydroxymethyltransferase 2 (SHMT2), suppresses the mitochondrial folate cycle, and consequently inhibits de novo purine synthesis [67].

IMP serves as the branch-point intermediate for de novo purine biosynthesis, functioning as the precursor for both adenosine monophosphate (AMP) and guanosine monophosphate (GMP). Conversion of IMP to AMP proceeds through the formation of adenylosuccinate, catalysed by adenylosuccinate synthetase (ADSS) using aspartate and guanosine triphosphate (GTP) as co-substrates, followed by enzymatic reaction of adenylosuccinate lyase (ADSL). Meanwhile, conversion to GMP also requires two sequential enzymatic steps: first, oxidation of IMP to xanthine monophosphate (XMP) by inosine monophosphate dehydrogenase (IMPDH), followed by amidation of XMP to GMP, by GMP synthetase (GMPS) using glutamine as the nitrogen donor (Fig. 2). In SCLC, ASCL1^low^-MYC^high^ cells exhibit markedly elevated levels of guanosine nucleotides and significant upregulation of *IMPDH1* and *IMPDH2* compared to ASCL1^high^ tumours. Functional studies show that IMPDH inhibition reduces RNA polymerase I-dependent pre-rRNA transcription and strongly suppresses the growth of ASCL1^low^ cells in vitro, while also selectively limiting the growth of ASCL1^low^ xenografts. This vulnerability appears to arise because de novo GTP is required for RNA polymerase I localization to ribosomal DNA [68, 69]. Of note, the expression of *IMPDH* is transcriptionally regulated by MYC, linking guanosine nucleotide production directly to oncogenic signalling [70, 71]. A similar dependence on guanosine metabolism has also been described in ALK^+^ LUAD, where ALK phosphorylates and activates guanylate kinase 1 (GUK1), thereby enhancing GDP/GTP biosynthesis and promoting tumourigenesis and aggressive tumour growth [72].

As de novo purine synthesis is a costly process that requires not only six ATP per synthesised purine but also 10 catalytic reactions, cancer cells benefit from nucleotide salvage alongside the de novo pathway. Salvage of catabolised nucleotides or nucleobases obtained from diet is a cost-effective process, requiring only one ATP molecule per synthesized purine. In the purine salvage pathway, adenine phosphoribosyltransferase (APRT) salvages free adenine by transferring the phosphoribosyl group from PRPP to generate AMP, which can then be deaminated to form IMP. The second key enzyme, hypoxanthine-guanine phosphoribosyltransferase (HPRT1), catalyses the PRPP-dependent salvage of hypoxanthine to IMP and guanine to GMP, thereby maintaining purine nucleotide pools and supporting nucleotide homeostasis [73] (Fig. 2). In the lung, purine metabolism appears to be highly adaptable, with tumour cells shifting between salvage and de novo synthesis depending on nutrient availability and oncogenic context. In vivo tracer studies using ^15^N_5_-adenine and ^15^N_5_-adenosine showed that adenosine is rapidly and preferentially salvaged in the lung, leading to efficient labelling of AMP and IMP, whereas adenine is incorporated much more slowly. These labelling patterns suggest that salvaged adenosine can support AMP production either via adenine as an intermediate, or through deamination to inosine, followed by HPRT1-mediated formation of IMP [74]. This reliance on the salvage pathway appears to be particularly important in SCLC, where high *HPRT1* expression has been linked to larger tumour size and more aggressive disease, suggesting that tumour cells depend on salvaged purines when glutamine is limited [75]. A similar metabolic dependency has been reported in NSCLC under conditions of impaired mitochondrial respiration, when OXPHOS is compromised and cells become more glycolytic, the oxidative arm of the PPP is activated to generate PRPP, thereby sustaining purine salvage. In this setting, *HPRT1* upregulation and increased IMP production become critical for continued proliferation [76]. By contrast, lung cancers carrying *KEAP1* mutations tend to depend more heavily on de novo purine synthesis, and this vulnerability can be exploited by glutamine antagonists such as DRP-104, which suppress tumour growth by limiting de novo purine biosynthesis [77].

Taken together, the differential dependence on, and most importantly, the remarkable plasticity between de novo and salvage purine synthesis pathways across diverse lung cancer subtypes underscores the adaptive capacity of cancer cells to rewire nucleotide metabolism in response to their metabolic needs and environmental cues, thereby ensuring sustained survival and uncontrolled growth [78].

### De novo and salvage metabolism of pyrimidines

Pyrimidine synthesis begins with glutamine, bicarbonate, and ATP, which are used by carbamoyl phosphate synthetase 2 (CPS2) to form carbamoyl phosphate. Next, aspartate transcarbamoylase (ATCase) transfers the carbamoyl group to aspartate to form N-carbamoyl-L-aspartate, which is then converted by dihydroorotase (DHO) to dihydroorotate. In mammals, CPS2, ATCase, and DHO are domains of the multifunctional CAD protein. Subsequently, dihydroorotate is oxidised by mitochondrially localised dihydroorotate dehydrogenase (DHODH) to orotate. Orotate is then attached to PRPP to produce orotidine 5’-monophosphate (OMP), which is subsequently converted to uridine monophosphate (UMP) by UMP synthetase (UMPS). From UMP, cells generate uridine diphosphate (UDP) and uridine triphosphate (UTP). The latter can be aminated by cytidine triphosphate synthetase (CTPS) to cytidine triphosphate (CTP). In the deoxyribonucleotide branch, UDP is converted through a series of steps into dTTP, which is ultimately used for DNA synthesis [79, 80] (Fig. 2). The subcellular localisation of DHODH in the inner mitochondrial membrane, together with its dependence on ubiquinone as an electron acceptor within the ETC, places mitochondria at a central regulatory position in pyrimidine synthesis. This mitochondrial dependency is not only biochemically relevant but also functionally important in cancer, as DHODH has been shown to be indispensable for SCLC survival and growth; upon its inhibition and stalling of the de novo route, these cells are unable to efficiently rely on the salvage pathway, unlike LUAD cells [81].

Consistent with the central role of mitochondria in the pyrimidine pathway, CPS1, a mitochondrially localized and rate-limiting enzyme of the urea cycle, has been demonstrated to be essential for the survival and proliferation of EGFR- and liver kinase B1 (LKB1)-deficient NSCLC cells [82]. Under normal physiology, CPS1 channels excess nitrogen by converting ammonia and bicarbonate into carbamoyl phosphate for ureagenesis; however, in these cancer cells, the generated carbamoyl phosphate is preferentially diverted into de novo pyrimidine biosynthesis, where it supports the production of pyrimidine nucleotides required for DNA synthesis and cell-cycle progression. Accordingly, *CPS1* suppression reduces tumour growth not by causing toxic ammonia accumulation, but by depleting pyrimidine nucleotide pools and thereby limiting nucleotide availability for replication [82–84]. The discovery of H3B-120 as a selective CPS1 inhibitor highlights the enzyme as a promising therapeutic target, although further in vivo validation is needed to establish its full translational potential [85].

Beyond these metabolic enzymes, the B-cell lymphoma 2 (BCL-2) protein family also links mitochondria to pyrimidine metabolism. As a member of the BCL-2 protein family, BCL-2-related ovarian killer (BOK), although classically regarded as a proapoptotic protein, has more recently been shown to perform important cell death-independent functions. In particular, BOK positively regulates UMPS, a key enzyme in de novo pyrimidine synthesis [86], and its loss reduces proliferation in NSCLC cell lines by lowering UMP levels and causing a consequent pyrimidine/purine nucleotide imbalance [87] (Fig. 2). Consistent with this metabolic disturbance, *BOK* knockout is accompanied by increased expression of cell-cycle regulators such as p53 and p21 [86], and later work demonstrated that the proliferative defect caused by BOK loss in KRAS-deficient lung cancer cells is entirely p53-dependent [88]. Loss of BOK has been shown to increase baseline DNA damage in NSCLC cells. In p53-proficient cells, this damage activates p53-dependent checkpoint arrest, whereas in p53-deficient cells the protective response is lost, leaving the cells reliant on ataxia telangiectasia mutated and rad3-related (ATR)-mediated DNA repair and rendering them selectively sensitive to ATR inhibition. Importantly, the DNA-damage phenotype can be rescued by a cell-permeable BOK-BH3 peptide, confirming that the metabolic effect is directly linked to BOK function rather than a nonspecific stress response. Together, these findings position BOK as a context-dependent regulator of pyrimidine metabolism and genome stability, whose loss can impair proliferation through p53 when the checkpoint is intact, but create a therapeutically exploitable ATR dependency when p53 is absent [87].

Given the central role of mitochondria and the rate-limiting enzyme DHODH in de novo pyrimidine synthesis, cancer cells often rely on pyrimidine salvage to maintain nucleotide homeostasis and reduce the energetic burden associated with de novo nucleotide production. In this pathway, uridine-cytidine kinase (UCK) catalyses the phosphorylation of uridine to UMP and cytidine to CMP, while cytidine deaminase (CDA) converts cytidine to uridine via hydrolytic deamination, and uridine phosphorylase (UPP) further regulates uridine homeostasis by reversibly converting uridine to uracil and ribose-1-phosphate. In addition, thymidine salvage contributes to nucleotide pool maintenance, as thymidine kinase (TK) phosphorylates thymidine to thymidine monophosphate (dTMP), which is subsequently converted through sequential phosphorylation to deoxythymidine triphosphate (dTTP) [73, 80].

The predominantly cytosolic uridine-cytidine kinase 2 (UCK2) has emerged as a potential early diagnostic biomarker in lung cancer, as its elevated expression is detectable in early-stage disease and is associated with adverse pathological features, poorer prognosis, and reduced overall survival in both NSCLC and SCLC [89]. In LUAD, UCK2 directly interacts with GTPase Ras homolog enriched in brain (RHEB), thereby activating mechanistic target of rapamycin (mTOR) signalling and its downstream pro-metastasis and proliferation signalling networks [90]. In ALK^+^-NSCLC, *CDA* is upregulated in ceritinib-resistant cells, as a result of hypomethylation. This highly proliferative and EMT-like CDA^high^ cells represent a small subpopulation within treatment-naïve tumours and are selectively enriched under therapy pressure. Treatment with modified DNA nucleosides such as 5-formyl-2′-deoxycytidine, selectively causes DNA damage in CDA^high^ resistant cells and shows therapeutic potential [91]. Uridine phosphorylase 1 (UPP1) has emerged as an important contributor to LUAD progression through its association with an immunosuppressive tumour microenvironment. UPP1 has been linked to LUAD progression through a tumour cell program characterized by preferential localization at the invasive front, enrichment in aggressive disease, and association with an immune-suppressive stromal context. UPP1^high^ tumour cells exhibit increased cytokine production, particularly transforming growth factor β1 (TGF-β1), and promote programmed death ligand 1 (*PD-L1*) expression via PI3K/AKT/mTOR signalling, supporting immune evasion and a tumour-promoting microenvironment [92]. Consistent with its metabolic role, UPP1 can also divert uridine-derived ribose into glycolysis under nutrient-limited conditions, thereby supporting central carbon metabolism when glucose availability is restricted [93]. The cytoplasmic isoform of TK (TK1), is significantly upregulated in LUAD in comparison to healthy lungs, contributing to poor clinical outcomes and higher relapse. Loss of TK1 reduces tumour growth and metastasis by altering GTP/GDP ratio and thereby hindering Rho GTPase activity [94]. Higher pre-treatment serum TK1 activity independently correlated with inferior survival in NSCLC and SCLC, while even less than two-fold increases during the first chemotherapy cycles were linked to poor treatment response [95].

In summary, mitochondrial pathways are central coordinators of de novo pyrimidine metabolism, integrating nitrogen handling, electron transport, and nucleotide biosynthesis to support DNA synthesis and cell proliferation. To circumvent this dependency, cancer cells can adapt by engaging pyrimidine salvage pathways, reflecting a high degree of metabolic flexibility. The exploitation of urea cycle and the use of uridine-derived ribose to fuel glycolysis illustrates how cancer cells repurpose core metabolic functions to sustain growth. Despite this adaptability, the mitochondrial dependence of de novo pyrimidine synthesis remains a critical vulnerability in lung cancer, which combined with strategies that disrupt salvage pathways may offer therapeutic opportunities that exploit the metabolic and DNA-repair constraints of tumour cells.

## The interplay of serine and glycine in one-carbon metabolism and redox balance

Serine and glycine are central to the biosynthetic and redox demands of proliferating tumour cells. Serine contributes to protein synthesis, nucleotide production, and antioxidant defence, while its interconversion with glycine and one-carbon units supports folate-dependent reactions required for DNA synthesis, methylation, and cellular repair. Glycine, in turn, is not only a product of serine metabolism but also an important precursor of GSH, helping tumour cells maintain redox homeostasis. Since both serine and glycine can be synthesized internally and be imported, tumour cells gain metabolic flexibility that helps them adapt to fluctuating nutrient availability [96].

**Fig. 3:**
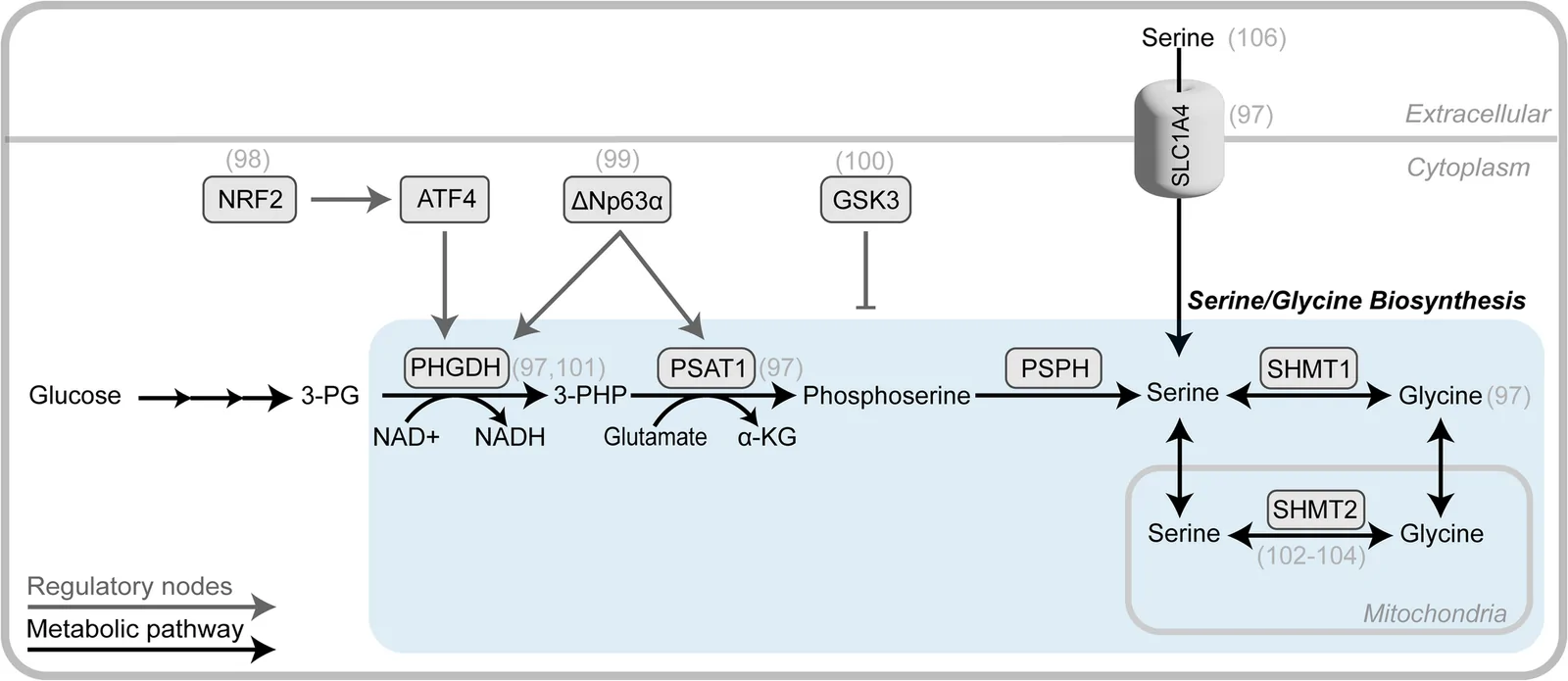
De novo serine and glycine biosynthesis pathway. Branched from 3-PG, an intermediate in glycolysis, the serine biosynthesis pathway proceeds through consecutive enzymatic reactions catalysed by PHGDH, PSAT1, and PSPH. The generated serine can then be converted to glycine in the cytoplasm and mitochondria by SHMT1 and SHMT2, respectively (blue). Key regulators such as NRF2 and ΔNp63α positively modulate the pathway, while GSK3 suppresses its regulatory nodes. In parallel, serine transporters help maintain intracellular serine pools as a compensatory mechanism supporting the de novo pathway 3-PG: 3-phosphoglycerate; ATF4: activating transcription factor 4; GSK3: glycogen synthase kinase 3; NRF2: nuclear factor erythroid 2-related factor 2; PHGDH: phosphoglycerate dehydrogenase; PSAT1: phosphoserine aminotransferase 1; PSPH: phosphoserine phosphatase; SHMT1/2: serine hydroxymethyltransferase 1 and 2; SLC1A4: solute carrier family 1 member 4; ΔNp63α: delta Np63 alpha

The de novo serine synthesis pathway represents a glycolysis-derived metabolic branch that diverts carbon from 3-phosphoglycerate (3-PG) into serine production. This pathway consists of three consecutive enzymatic steps. First, phosphoglycerate dehydrogenase (PHGDH) catalyses the oxidation of 3-PG to 3-phosphohydroxypyruvate (3-PHP), accompanied by NADH generation. Next, phosphoserine aminotransferase 1 (PSAT1) mediates a transamination reaction in which an amino group from α-KG is transferred to 3-PHP, forming phosphoserine. In the final step, phosphoserine phosphatase (PSPH) dephosphorylates phosphoserine to yield serine. Following its synthesis, serine can be further metabolized into glycine, with SHMT1 functioning in the cytosol and SHMT2 operating within mitochondria (Fig. 3).

^13^C-glucose tracing studies have shown that a subset of NSCLC cell lines can synthesize glycine from serine. These cells also show marked upregulation of *PHGDH*, the rate-limiting enzyme in serine biosynthesis [97]. More broadly, *PHGDH* and other serine pathway components, including *PSAT1* and the serine transporter *SLC1A4*, are frequently overexpressed in lung cancer, especially in LUSC, where high expression is linked to poorer survival. Similar heterogeneity has been described in LUAD, where a PHGDH^high^ subtype shows a more aggressive phenotype, with advanced stage, greater lymph node metastasis, and significantly worse five-year overall survival than PHGDH^low^ tumours [97]. At the transcriptional level, *PHGDH* is regulated by the NRF2-activating transcription factor 4 (ATF4) axis, with NRF2 promoting serine biosynthesis gene expression through ATF4-dependent promoter activation [98]. In LUSC, the lineage-associated transcription factor ΔNp63α provides an additional layer of control by directly activating *PHGDH*, *PSAT1*, and *SLC1A4*. Notably, *ΔNp63α* knockdown reduces both ATF4 and NRF2 protein levels, suggesting coordination between these programs [99]. In parallel, nuclear glycogen synthase kinase 3β (GSK3β) acts as a transcriptional suppressor of *PHGDH*, *PSAT1*, *PSPH*, and *SHMT2* [100]. Together, these regulatory inputs shape a high-serine state in which PHGDH^high^ NSCLC tumours preferentially channel serine-derived carbons into GSH and pyrimidine synthesis, supporting rapid proliferation and enhanced metabolic flux [97]. Beyond these metabolic effects, PHGDH also plays a key role in lung metastatic colonization. Lung metastases depend on PHGDH-driven serine biosynthesis to activate mechanistic target of rapamycin complex 1 (mTORC1) signalling. In the lung microenvironment, abundant pyruvate is available to support serine synthesis, generating cytosolic α-KG as a by-product, which in turn promotes mTORC1 phosphorylation and protein translation. This lung-specific adaptation renders PHGDH-expressing metastases sensitive to mTORC1 inhibition by rapamycin, whereas *PHGDH*-silenced tumours lose this dependency [101]. Thus, PHGDH functions as a dual hub in lung cancer: it supports redox balance and nucleotide synthesis through serine/glycine metabolism, thereby defining a clinically aggressive metabolic state with poor prognosis, and it also promotes metastatic growth in the lung by linking pyruvate availability to mTORC1-driven proliferation.

The mitochondrial conversion of serine to glycine by SHMT2 supports DNA repair and recovery after radiotherapy in NSCLC cells. Following irradiation, serine and glycine levels decrease in both patient plasma and NSCLC cell lines, reflecting their active consumption for dTTP synthesis, which is required for efficient DNA repair and post-radiation recovery. This response appears to be tumour-selective, as it is not observed in adjacent healthy tissue. Accordingly, combined inhibition of SHMT2 and radiotherapy suppresses NSCLC cell growth by increasing ROS and oxidative damage [102]. This SHMT2 dependency also extends to nutrient stress; when glucose is limited, NSCLC cells enhance de novo serine synthesis and strengthen serine-to-glycine conversion through SHMT2, thereby preserving one-carbon flux for redox control and biosynthesis [103]. In KEAP1/LKB1-deficient KRAS-mutant lung cancer, this reliance is further intensified, as LKB1 loss enhances SHMT-driven serine-glycine/one-carbon metabolism and links it to antioxidant defence, rendering these tumours especially sensitive to SHMT inhibition [104]. Together, these findings position SHMT1/2 as a central component of a stress-adaptive network that supports DNA repair, redox homeostasis, and metabolic flexibility in lung cancer. This metabolic flexibility is further illustrated by NSCLC cell lines cultured in Plasmax [105], a medium designed to more closely resemble physiological nutrient conditions than standard media such as DMEM or RPMI. Under the lower serine availability in Plasmax, the cells decrease their dependence on extracellular serine by increasing de novo serine synthesis and diverting hypoxanthine toward purine biosynthesis. This rerouting allows serine to be conserved for other essential functions, including GSH production, rather than being used predominantly for nucleotide synthesis. Notably, this adaptation does not substantially alter total purine nucleotide pools or cell proliferation [106].

Overall, serine and glycine metabolism emerge as flexible support systems that help lung cancer cells meet changing demands for growth, survival, and adaptation. By linking nutrient use to biosynthesis, redox control, and signalling, this pathway helps tumours sustain proliferation under stress and contributes to more aggressive disease behaviour. In this way, serine/glycine metabolism represents not only a metabolic adaptation but also a potential therapeutic vulnerability.

## The role of sulphur metabolism in oxidative balance and cellular adaptation

Sulphur metabolism plays a central role in cellular homeostasis by sustaining the availability of biologically active sulphur for essential metabolic processes. Through its contributions to redox balance, thiol maintenance, and methylation-related pathways, it helps preserve cellular integrity under both basal and stress conditions. Sulphur-dependent metabolites also support adaptive responses to oxidative challenge and metabolic fluctuation, placing sulphur metabolism at the intersection of biosynthesis, signalling, and stress resilience.

**Fig. 4:**
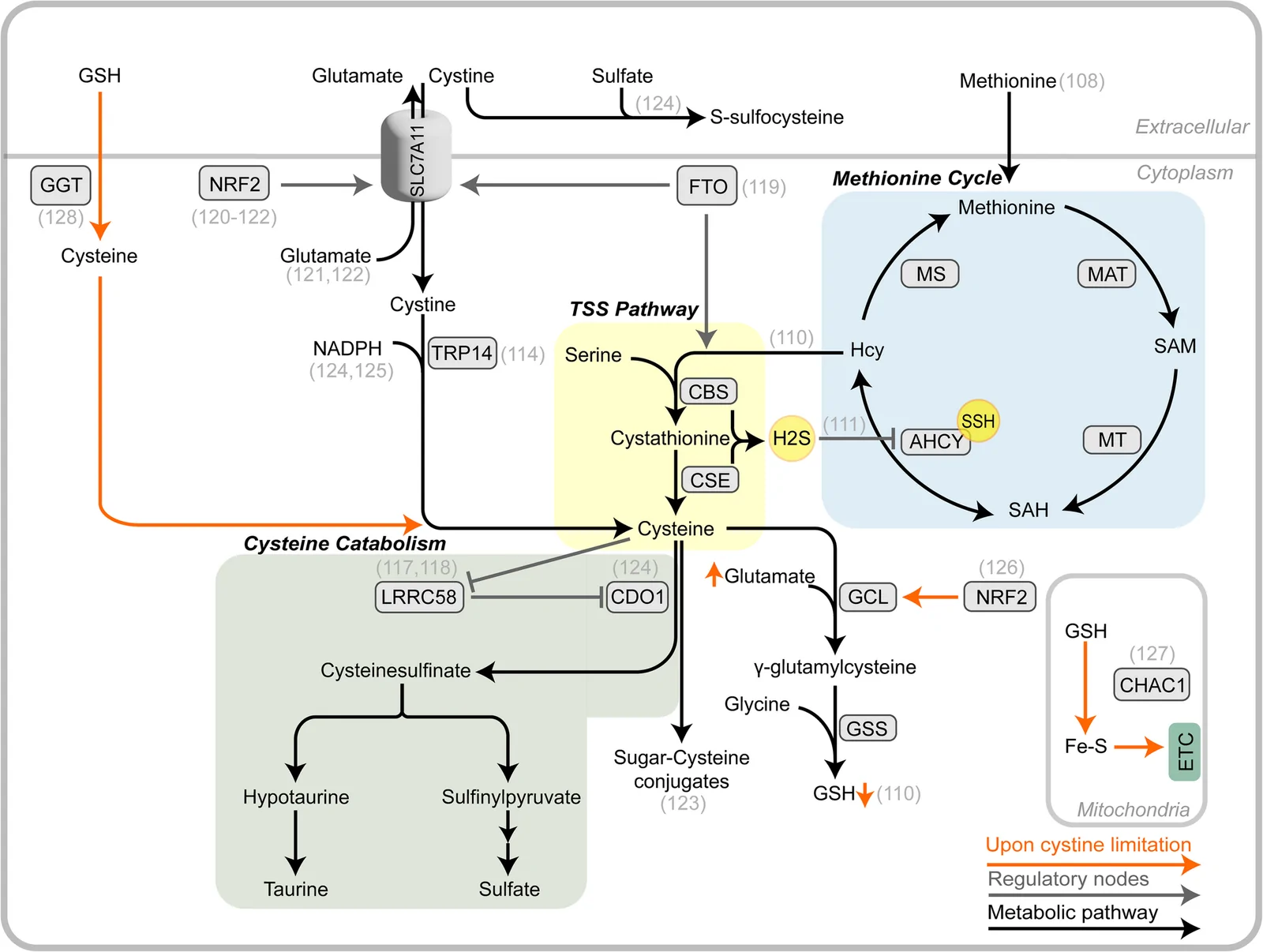
Sulphur-related metabolic pathway. The intracellular pool of cysteine is replenished either by dietary methionine, which enters the methionine cycle (blue) and then funnels the sulphur-containing Hcy into TSS (yellow), or via import of extracellular cystine in exchange for glutamate efflux. Cysteine is then used for GSH biosynthesis, which is crucial for maintaining oxidative balance. However, when cysteine accumulates, the catabolic arm of sulphur metabolism is activated by CDO1 (green). Alternatively, surplus cysteine can form conjugates with glucose-derived sugars. When cysteine is scarce, extracellular and intracellular GSH are degraded to restore sulphur supply, while excess glutamate is converted into γ-glutamyl peptides to prevent its toxic accumulation. NRF2 and FTO are dominant regulators of the pathway at multiple levels AHCY: adenosylhomocysteinase; CBS: cystathionine beta-synthase; CDO1: cysteine dioxygenase 1; CHAC1: chaC glutathione specific gamma-glutamylcyclotransferase 1; CSE: cystathionine gamma-lyase; ETC: electron transport chain; Fe-S: iron-sulfur; FTO: fat mass and obesity-associated protein; GCL: glutamate-cysteine ligase; GGT: gamma-glutamyltransferase; GSH: glutathione; GSS: glutathione synthetase; H2S: hydrogen sulfide; Hcy: homocysteine; LRRC58: leucine-rich repeat-containing protein 58; MAT: methionine adenosyltransferase; MS: methionine synthase; MT: methyltransferase; NADPH: nicotinamide adenine dinucleotide phosphate, reduced form; NRF2: nuclear factor erythroid 2-related factor 2; SAH: S-adenosylhomocysteine; SAM: S-adenosylmethionine; SLC7A11: solute carrier family 7 member 11; SSH: hydropersulfide; TRP14: thioredoxin reductase 1-like protein 1

Methionine is an essential sulphur-containing amino acid that is obtained from the diet and funnelled into the methionine cycle, where methionine adenosyltransferases (MATs) convert it into S-adenosylmethionine (SAM) in an ATP-dependent reaction. SAM serves as the universal methyl donor for methyltransferase (MT) reactions, supporting methylation of DNA, RNA, proteins, and histones, a reaction during which S-adenosylhomocysteine (SAH) is produced. SAH is then hydrolysed by adenosylhomocysteinase (AHCY, also known as SAHH) to generate homocysteine (Hcy) in a reversible manner. At this step, Hcy is either remethylated, for example by betaine as the methyl donor, to regenerate methionine or enters the TSS pathway [107] (Fig. 4). Tumour-initiating cells of the lung appear to be reliant on methionine for their maintenance and identity, as they exhibit high methionine cycling flux and a correspondingly elevated demand for transmethylation. In this state, methionine consumption outpaces the capacity for remethylation, rendering endogenous recycling insufficient to sustain intracellular methionine and SAM pools. As a result, tumour-initiating cells become reliant on exogenous methionine to preserve methyl-donor availability, maintain epigenetic regulation, and support their tumourigenic program. Disruption of methionine metabolism therefore compromises their initiating capacity, highlighting methionine as a bona fide metabolic dependency rather than a permissive nutrient [108].

At the crossroad of Hcy metabolism, sulphur transfer through TSS is initiated by cystathionine β-synthase (CBS), which condenses Hcy and serine into cystathionine. This reaction is followed by cleavage and hydrolysis of cystathionine by cystathionine γ-lyase (CSE, also called cystathionase, CTH) into cysteine, α-ketobutyrate, and ammonium [109] (Fig. 4). Stable isotope tracing showed that de novo cysteine synthesis is largely absent in SCLC and NSCLC cell lines, suggesting a limitation in the TSS pathway at the level of CBS and CSE. Consistent with this, lung tumours display subtype-specific remodelling of these enzymes; NSCLC tumours generally exhibit lower *CBS* and higher *CSE* expression relative to normal lung, whereas SCLC tumours show the opposite pattern [110]. Cysteine, the second sulphur-containing amino acid, is used as a precursor for GSH biosynthesis in a two-step reaction [109]. In the first and rate-limiting reaction, cysteine is combined with glutamate by glutamate-cysteine ligase (GCL) to form γ-glutamylcysteine. Then, glycine is added by catalytic reaction of glutathione synthetase (GSS), and reduced GSH is formed. Beyond their canonical roles in TSS, CBS and CSE also contribute to non-canonical hydrogen sulphide (H_2_S) production. In NSCLC cells, H_2_S-mediated persulfidation of AHCY suppresses homocysteine generation, thereby limiting substrate entry into the TSS pathway and restricting de novo cysteine synthesis needed for GSH production and ferroptosis resistance. This regulatory mechanism can be further reinforced under cystine starvation to ensure complete depletion of cysteine pools [111].

Apart from de novo synthesis of cysteine via TSS, its oxidised form, cystine, can be taken up from the microenvironment by the SLC7A11 (xCT) antiporter in exchange for glutamate, in order to replenish the intracellular cysteine pool [112, 113]. Upon uptake, cystine is reduced by thioredoxin-related protein of 14 kDa (TRP14), in an NADPH-dependent manner, to form cysteine [114] (Fig. 4). Intracellular cysteine levels are maintained through tight, concentration-dependent control. One key catabolic pathway is mediated by cysteine dioxygenase 1 (CDO1), which irreversibly oxidizes surplus cysteine to cysteinesulfinate [115]. Cysteinesulfinate can then be metabolized either to sulfinylpyruvate, which is ultimately converted to sulphate for excretion, or to hypotaurine and then taurine [116]. The activity and stability of CDO1 are regulated by leucine-rich repeat-containing protein 58 (LRRC58) according to cysteine availability. When cysteine is scarce, LRRC58 promotes proteasomal degradation of CDO1, thereby conserving the limited cysteine pool for essential cellular processes rather than diverting it toward sulphate or taurine production. When cysteine levels rise, LRRC58 activity is reduced, CDO1 is stabilized, and cysteine catabolism is enhanced [117, 118] (Fig. 4).

In NSCLC, the fat mass and obesity-associated protein (FTO), an m6A RNA demethylase, acts as an epitranscriptomic regulator of cysteine metabolism. FTO promotes expression of *SLC7A11*, *CBS*, and *CSE* by reducing m6A methylation on their transcripts, thereby sustaining both extracellular cystine uptake and de novo cysteine synthesis. Genetic or pharmacologic inhibition of FTO reduces *Slc7a11*/*Cbs*/*Cse* expression, depletes GSH, raises ROS and DNA damage, and suppresses tumour growth, identifying the FTO-SLC7A11-CBS-CSE axis as a targetable metabolic vulnerability in lung cancer [119]. NRF2 further enhances cystine uptake by upregulating *SLC7A11*, creating a distinctive metabolic signature that can be non-invasively detected using positron emission tomography of the system xc⁻ pathway, [¹⁸F]FSPG. This imaging approach enables reliable identification of NRF2-activated NSCLC subtypes across orthotopic models and patient-derived tumours [120]. In KEAP1-mutant NSCLC cells, chronic NRF2 activation sustains high cystine influx and glutamate efflux, thereby supporting glutathione biosynthesis while depleting intracellular glutamate pools required for anaplerosis and central carbon metabolism [120–122]. This metabolic rewiring increases dependence on glutamine catabolism, making NRF2/KEAP1-altered cells particularly vulnerable to glutaminase inhibition [121, 122]. However, imported cystine is not exclusively directed toward GSH synthesis. In NRF2-activated LUAD and LUSC, a substantial fraction of cystine is diverted into non-canonical cysteine-derived products, including conjugates formed with glucose-derived sugar metabolites. In these cells, high cystine uptake together with elevated intracellular free cysteine is associated with reduced proliferative capacity, indicating that growth inhibition is driven by cysteine stress and conjugate accumulation rather than by glutamate export alone [123]. In parallel, these increased intracellular cysteine levels induce CDO1 accumulation, likely as a compensatory response that channels excess cysteine into oxidative catabolism and downstream taurine and/or sulphite production through cysteinesulfinate. The resulting sulphate can further deplete extracellular cystine by cleaving its disulphide bond and forming S-sulfocysteine. In NSCLC, however, *CDO1* is frequently silenced by promoter methylation, presumably to prevent excessive cysteine depletion and the accumulation of potentially toxic sulphur metabolites. Re-expression of functional *CDO1* in NSCLC cells suppresses proliferation, at least in part by lowering glutathione levels and raising sulphate to cytotoxic concentrations [124]. In these cells, the continual reduction of imported cystine to cysteine consumes intracellular NADPH, thereby increasing reliance on the PPP and its NADPH-generating output to preserve redox homeostasis. This creates a vulnerability under glucose-limited conditions, as glucose fuels the PPP [124]. Accordingly, GLUT inhibition showed therapeutic potential in tumours with high *SLC7A11* expression [125].

Cystine depletion triggers ferroptotic cell death in most lung cancer cells, as they remain heavily dependent on extracellular cystine to sustain GSH synthesis [110] (Fig. 4). Apart from that, toxic accumulation of glutamate has been shown to contribute to their death. To counter this stress, NRF2 induces the catalytic subunit of GCL, which diverts glutamate into γ-glutamyl peptide synthesis and thereby limits glutamate overload [126]. As an additional adaptive mechanism to cystine starvation, NSCLC cells catabolize mitochondrial GSH through gamma-glutamyl cyclotransferase (CHAC1) to help replenish sulphur-containing metabolites and preserve the function of iron-sulphur-cluster (Fe-S)-containing proteins in the mitochondrial ETC, thereby sustaining respiratory activity [127]. Extracellular glutathione can also serve as an alternative amino acid source under cysteine-limited conditions, and its breakdown by γ-glutamyltransferases (GGT) provides tumours with cysteine and other nutrients, rather than functioning solely as a redox buffer [128].

Overall, these findings indicate that sulphur metabolism is not merely a supportive pathway, but a major determinant of tumour cell fitness. Across lung cancer models, the sulphur axis is repeatedly rewired to secure methyl-donor supply, maintain cysteine and glutathione homeostasis, and protect against oxidative and ferroptotic stress. At the same time, these adaptations create metabolic liabilities by increasing dependence on extracellular nutrients and specific redox-supportive pathways. Taken together, sulphur metabolism emerges as a central node linking biosynthesis, stress adaptation, and therapeutic vulnerability in lung cancer.

## Clinical translation of metabolic vulnerabilities in lung cancer: current status and future directions

In advanced lung cancer, the bulk of interventional studies still focus on blocking oncogenic signalling nodes such as *EGFR* and *ALK*, or the downstream *PI3K/AKT/mTOR* cascade, with metabolic rewiring treated as a consequence rather than the primary target. Agents include second- and third-generation EGFR tyrosine kinase inhibitors (gefitinib, erlotinib, afatinib, osimertinib), ALK inhibitors (crizotinib, ceritinib, alectinib, brigatinib, repotrectinib), and antibody-drug conjugates [129, 130]. These drugs are typically evaluated either as monotherapies in genomically defined subsets or in combinations such as anlotinib plus osimertinib or repotrectinib plus osimertinib, aiming to deepen blockade of receptor tyrosine kinase signalling and its proliferative and survival outputs. In parallel, immunotherapies targeting PD-1/PD-L1 have become central in both NSCLC and SCLC, where standard regimens still rely heavily on platinum-etoposide chemotherapy [131]. These cytotoxic and immune-based approaches primarily act through DNA damage and immune modulation, with any metabolic effects considered secondary rather than being designed as explicit metabolic interventions.

Among metabolism-focused interventions, glycolysis-targeting strategies are the most developed. A key clinical example is 2-deoxy-D-glucose (2DG), a glucose analogue that enters cells via GLUTs, is phosphorylated by hexokinase II, and then stalls, thereby blocking further glycolytic processing. This mechanism is expected to preferentially stress highly glycolysis-dependent and hypoxic tumour cells by limiting ATP generation and reducing their ability to support survival under metabolic stress, thereby potentially enhancing sensitivity to cytotoxic therapy. In a phase I dose-escalation trial in advanced solid tumours including lung cancer, 2DG was administered alone and in combination with docetaxel to define safety, pharmacokinetics, dose-limiting toxicities, and exploratory efficacy [132]. Closely related approaches target glycolysis without a dedicated tumour‑cell glycolytic inhibitor, either by interrogating glucose metabolism as a biomarker or by modulating systemic glucose availability. In NSCLC, a fludeoxyglucose-F-18 positron emission tomography (FDG‑PET)-based imaging study evaluates how targeted agents (such as EGFR inhibitors or hormonal modulators), in combination with chemotherapy, reshape tumour glucose uptake, correlating dynamic FDG‑PET metrics with progression‑free and overall survival [133]. In a more host‑directed axis, a trial in lung cancer patients with comorbid diabetes, tests whether adding the sodium-glucose cotransporter‑2 (SGLT2) inhibitor, dapagliflozin, to standard oncologic care improves survival while carefully monitoring cardiovascular and metabolic safety [134]. Mechanistically, dapagliflozin reduces systemic glucose levels via renal SGLT2 inhibition, thereby indirectly restricting the glucose pool accessible to tumour cells, in contrast to glycolytic inhibitors such as 2DG.

Downstream of glycolysis, mitochondrial pyruvate handling has been targeted with dichloroacetate (DCA), which inhibits pyruvate dehydrogenase kinase 1 (PDK1) and thereby activates pyruvate dehydrogenase. This shifts pyruvate away from lactate production and toward mitochondrial oxidation. Clinically, DCA has been evaluated in a phase II trial in previously treated metastatic NSCLC patients to assess safety and early activity [135]. Like 2DG, DCA is intended to exploit tumour dependence on aerobic glycolysis, but it acts at a later step in the pathway; 2DG interferes with glycolytic entry and processing, whereas DCA redirects pyruvate into mitochondrial metabolism.

Targeting glutamine catabolism and mTOR‑dependent anabolism represents another metabolic branch now in clinical testing. The glutaminase inhibitor CB‑839 (telaglenastat) has been evaluated broadly in solid tumours, including NSCLC, to block glutamine‑driven anaplerosis into the TCA cycle [136]. Building on this, an NSCLC‑specific trial combines CB‑839 with the dual mTORC1/2 inhibitor sapanisertib (MLN0128) in advanced disease, aiming to simultaneously restrict glutamine‑derived carbon entry into mitochondria and suppress mTOR‑regulated anabolic and survival signalling [137]. These studies extend metabolic targeting beyond glucose, probing whether coordinated inhibition of glutamine metabolism and growth‑factor signalling can create a more durable antitumour effect. In parallel, although mesothelioma represents a distinct thoracic malignancy, it is noteworthy that mitochondrial metabolism is also being therapeutically targeted in this context. Specifically, RSO-021, a first-in-class covalent inhibitor of the mitochondrial peroxiredoxin 3 (PRX3), is currently under investigation in the ongoing MITOPE Phase 1/2 clinical trial [138].

Taken together, current evidence indicates that lung cancer clinical research is still dominated by gene- and protein-centric interventions, whereas direct targeting of tumour metabolism remains confined to a relatively small, predominantly early-phase segment of the therapeutic portfolio. Within this metabolic space, most clinical activity clusters around two major axes, glucose and glutamine metabolism, with trials testing glycolysis modulators, mitochondrial pyruvate-directed agents, and glutaminase/mTOR-targeting combinations in selected NSCLC populations. In contrast, SCLC has essentially no clinical trials explicitly designed around metabolic vulnerabilities, even though preclinical work over the past years has increasingly focused on its metabolic dependencies. Collectively, these patterns underscore that metabolism-centric therapies in lung cancer, and particularly in SCLC, remain an emerging and underexploited area of translational research, with substantial room to expand beyond the current emphasis on oncogenic signalling and immune checkpoints.

## Concluding remarks

In conclusion, the metabolic alterations observed in lung cancer are unlikely to be explained by changes in a single pathway. Rather, malignant progression is supported by coordinated rewiring across interconnected metabolic circuits, including glucose, glutamine, lipid, nucleotide, and redox metabolism, which together enable cancer cells to preserve bioenergetic output, sustain biomass accumulation, and adapt to environmental and therapeutic stress. This integrated view helps explain why targeting a single pathway often produces only partial responses and why metabolic plasticity remains a major barrier to durable treatment benefit. We argue that the field’s most actionable gap is not the absence of targets, but the absence of patient-selection tools and of combinations designed to anticipate, rather than merely react to, metabolic plasticity. Functional imaging, including the recently validated [¹⁸F] FSPG PET tracer, offers a critical route to metabolic stratification, while metabolomic profiling of plasma or tumour interstitial fluid can complement imaging by resolving pathway activity that imaging alone cannot capture. Addressing these gaps will be essential for moving beyond pathway-centric interventions toward rational combination strategies and biomarker-guided metabolic therapies that better reflect the complexity of lung cancer biology.

## Data Availability

Not applicable.
